# The significance of upfront autologous stem cell transplantation for high‐intermediate/high‐risk stage IV diffuse large B‐cell lymphoma

**DOI:** 10.1002/cnr2.1786

**Published:** 2023-02-28

**Authors:** Aleksei K. Koviazin, Larisa V. Filatova, Ilia S. Zyuzgin, Anna S. Artemyeva, Ilia L. Poliatskin, Darya S. Burda, Stanislav A. Volchenkov, Svetlana S. Elkhova, Tatiana Yu. Semiglazova

**Affiliations:** ^1^ Department of Hematology and Chemotherapy with Intensive Care Unit NMRC of Oncology n.a. N.N.Petrov of MoH of Russia, Federal State Budgetary Institution “Petrov National Medical Cancer Research Centre” of the Ministry of Health of the Russian Federation Saint‐Petersburg Russian Federation; ^2^ Department of Innovative Methods in Therapeutic Oncology and Rehabilitation NMRC of Oncology n.a. N.N.Petrov of MoH of Russia, Federal State Budgetary Institution “Petrov National Medical Cancer Research Centre” of the Ministry of Health of the Russian Federation Saint‐Petersburg Russian Federation; ^3^ Department of Oncology Federal State Budgetary Educational Institution of Higher Education “North‐Western State Medical University named after I.I. Mechnikov” of the Ministry of Health of the Russian Federation Saint‐Petersburg Russian Federation; ^4^ Laboratory of Tumor Morphology Federal State Budgetary Institution “Petrov National Medical Cancer Research Centre” of the Ministry of Health of the Russian Federation Saint‐Petersburg Russian Federation

**Keywords:** autologous hematopoietic stem cell transplantation, diffuse large B‐cell lymphoma, high‐risk, stage IV

## Abstract

**Background:**

Diffuse large B‐cell lymphoma (DLBCL) is the most common (30%–35%) type of B‐cell lymphoma. Only about 60% of all newly diagnosed advanced‐stage DLBCL can be completely treated with x6 R‐CHOP. High‐dose chemotherapy (HDCT) followed by autologous hematopoietic stem cell transplantation in the first remission (upfront auto‐HSCT) can serve as an option to improve a prognosis in these patients.

**Aims:**

This trial aimed to improve prognosis in DLBCL by upfront auto‐HSCT.

**Methods and Results:**

A group of 105 patients: DLBCL NOS, age 18–65, stage IV, IPI ≥2, CR/PR after x6 R‐CHOP/DA‐EPOCH‐R from 2010 to 2019 at NMRC of Oncology named after N.N.Petrov of MoH of Russia was retrospectively analyzed. The HSCT group included patients with upfront HDCT followed by auto‐HSCT (*n* = 35). The control group included patients with non‐invasive follow‐up after induction (*n* = 70). Primary endpoint was progression‐free survival (PFS). Secondary endpoints were overall survival (OS), response rate and relapse rate. The 3‐year OS (*p* = .013) and 3‐year PFS (*p* = .033) were significantly higher in the HSCT group. The 3‐year OS was decreased by the occurrence of relapse (*p* ≤ .001) and weight loss (B‐symptom) (*p* = .04). DEL was the negative prognostic factor for 3‐year PFS in all patients (*p* = .001) and control group (*p* = .001). DA‐EPOCH‐R significantly increased the 3‐year PFS (*p* = .041).

**Conclusion:**

Upfront HDCT followed by auto‐HSCT can increase 3‐year OS and PFS and improve prognosis in DLBCL NOS, age 18–65, stage IV, IPI ≥2 patients.

## 1 INTRODUCTION

Diffuse large B‐cell lymphoma (DLBCL) is the most common (30–35%) type of B‐cell lymphoma with an incidence of about 5.6 per 100.000 men and women per year.^1^ This disease is heterogeneous, and various characteristics, including morphology, immunohistochemical marker expression, genetic profile, and clinical behavior, can influence the treatment result and prognosis.^2^

The standard treatment of DLBCL is R‐CHOP immunochemotherapy (ICT).^3^ Approximately 85% of patients achieve complete responses by this course. Among complete responders, about 25% will relapse. A half of the relapsed will not be candidates for high‐dose chemotherapy (HDCT) with autologous stem cell transplantation (auto‐HSCT), which is the second‐line standard of treatment in this case.^4^ Therefore, about 60% of all newly diagnosed advanced‐stage DLBCL can be completely treated by standard ICT.^5^

Upfront auto‐HSCT was studied as a way to improve outcomes as a consolidative treatment option in the pre‐rituximab era, but conflicting results were reported.^6^, ^7^, ^8^ In the largest Cochrane meta‐analysis (*n* = 3079), an increased number of CR and improvement of PFS in high‐risk DLBCL without any OS benefit were found.^9^ However, these findings were not proven by other meta‐analyses in the same period.^10^, ^11^

In the last 10 years, several RCT trials compared ICT and upfront auto‐HSCT. A slight improvement of 2‐year failure‐free survival (FFS) was achieved in the DLCL04 trial in DLBCL patients,^12^ and temporal improvement of 3‐year disease‐free survival (DFS) among aaIPI‐2 patients was reported in NCT00355199.^13^ However, several retrospective trials demonstrated significant improvement in overall survival (OS)^14^, ^15^ and progression‐free survival (PFS)^16^ in DLBCL patients with high LDH^14^ and ааIPI ≥2,^15^, ^16^ who were consolidated by auto‐HSCT.

Two meta‐analyses^17^, ^18^ were conducted to sum the results of RCT^12^, ^13^, ^19^, ^20^ and non‐randomized trials.^14^, ^15^, ^16^, ^21^, ^22^, ^23^ Both of them demonstrated only short‐term survival benefits in high‐risk patients.

Based on these results, we performed our retrospective trial to investigate the possible benefit of upfront auto‐HSCT in stage IV high‐intermediate/high‐risk DLBCL.

## 2 MATERIALS AND METHODS

### 2.1 Study design

Retrospective cohort single‐center trial.

### 2.2 Patient selection

The main inclusion criterion was biopsy‐confirmed CD20(+) DLBCL NOS, according to the WHO 2017 criteria. All patients were from 18 to 65 years old. According to Russian legislation, a majority is reached at the age of 18 and younger patients are treated according to pediatric protocols. The peak age was selected to better match previous RCT^12^, ^19^ and by the results of comprehensive geriatric assessment.^24^

All patients had stage IV and high‐intermediate/high IPI scores (IPI ≥2). Stage IV was assessed by Lugano classification.

All patients had to be treated with chemotherapy of x6 R‐CHOP or x6 DA‐EPOCH‐R and achieved complete (CR) or partial (PR) response, classified by Lugano Response Criteria by the end of treatment. If PET‐CT was unavailable, the response was classified by CT and biopsy.

A total of 516 patients with C83.3 treated from Jan 2010 to Dec 2019 at NMRC of oncology n.a. N.N.Petrov were analyzed to meet the inclusion criteria of this study. After that, exclusion criteria were applied.

Firstly, age‐mismatched (*n* = 136) patients were excluded. Secondly, patients (*n* = 114) without biopsy‐confirmed CD20 (+) DLBCL NOS were excluded. After the medical data analysis, patients without stage IV (*n* = 23), with CNS involvement (*n* = 10), HIV (*n* = 18), EBV (*n* = 1), IPI = 1 (*n* = 1), and synchronous or metachronous malignancies (*n* = 20) were excluded.

Then, patients with SD/PD (*n* = 27) on ICT, non R‐CHOP/DA‐EPOCH‐R ICT (*n* = 26), and with rituximab consolidation (*n* = 5) were excluded. Also, patients with radiotherapy (RT) consolidation (*n* = 38) were excluded.

**FIGURE 1:**
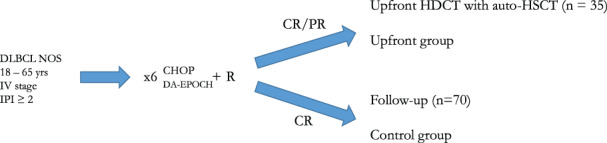
Study design.

In the end, we obtained 105 patients for further analyses (Figure 1).

The upfront group (*n* = 35) was combined from patients, who were assigned into HDCT with auto‐HSCT consolidation. The control group (*n* = 70) patients was on follow‐up without any consolidation after ICT. All inclusion decisions were approved by multidisciplinary team, confirmed by the institutional committee (Data S1).

Both “steady‐state” (filgrastim 10 μg/kg/day SC), and chemotherapy‐based protocols (DHAP ± R + filgrastim 5 μg/kg/day SC or cyclophosphamide 2 g/m^2^ IV + filgrastim 5 μg/kg/day SC) were used for stem cell mobilization.^25^ Stem cells were acquired from peripheral blood using apheresis through a central venous line.^26^ The conditioning regimen was LEAM ± R.^27^ Cryopreserved peripheral blood stem cells were infused on day 0. Supportive care was given according to institutional protocol.

Informed consent was obtained from the patients before data collection, following institutional guidelines, and the study was approved by the Committees for the Ethical Review of Research at NMRC of oncology n.a. N.N.Petrov.

### 2.3 Statistical analysis

The primary endpoint of this study was progression‐free survival (PFS, measured from the date of diagnosis to confirmed relapse or death from any cause). Secondary endpoints were overall survival (OS, measured from the date of diagnosis to death from any cause), response rate and relapse rate.

To determine differences in categorical variables (sex, age, DEL status, etc.) from independent samples, *χ* ^2^ with Yates correction or Fisher's exact test were used. In paired samples, McNemar criteria with Yates correction was used. Numerical variables (LDH, Ki67, etc.) were compared by Mann–Whitney test. Normality assumption check was done by Shapiro–Wilk test (Data S2).

Cox proportional hazards model was used to estimate the effects of the prognostic risk factors by univariate and multivariate settings with loss‐to‐follow‐up point censoring. Proportional hazards assumption was verified for all models. Only factors with *р* ≤ .1 in the univariate setting and Matthews correlation coefficient $<\left|0.3\right|$ were included in the multivariate setting (Data S3, Data S4). Kaplan–Meier method was used to estimate OS and PFS. Log‐rank test was used to evaluate differences in survival.

The sample size of the study protocol was estimated to test the difference in 3‐year PFS and relapse incidence between selected groups. The closest in design and inclusion criteria RCT with ours reported 13% relapse rate after upfront HDCT with auto‐HSCT.^13^ Relapse rate after ICT in target population is about 40%, according to the world data.^5^ A sample of 96 patients (upfront group *n* = 32, control group, *n* = 64) was required for a power of 80% and with a one‐sided *α* level of .05 with enrollment ratio 1:2.

A two‐sided *p*‐value of <.05 significance level was considered to be statistically significant in all analyses. All statistical analyses were performed using STATISTICA for Windows (ver. 12 Lic. №. BXXR310F964808FA‐V).

## 3 RESULTS

### 3.1 Patients and procedures characteristic

The two trial arms were well balanced. (Table 1) The mean age was 48.0 ± 11.7 years with a median of 53 (18–63) years in the upfront group and 48.9 ± 12.5 years with a median of 53 (22–64) years in the control group. The median observing period in the upfront group was 68 (19–82) months with a mean of 60.6 ± 21.2 months and 55 (6–143) months with a mean of 62.5 ± 41.1 months in the control group. Imbalances were found in bulky disease proportion (21/35 vs. 24/70, *p* = .02) only. There was a significantly higher proportion of R‐CHOP treated patients in the control group–79% (*n* = 55), *p* = .004. All numerical characteristics had non‐normal distribution (*р* < .001).

**TABLE 1:** Patient and procedures characteristics.

Characteristic	Criteria	Upfront, *n* = 35		Control, *n* = 70		*p*‐value
		*n*	%	*n*	%	
Sex	Male	18	51	32	46	.67
	Female	17	49	38	54	
ECOG	<2	30	86	58	83	.92
	≥2	5	14	12	17	
В‐symptoms	Yes	18	51	33	47	1.0
	No	17	49	37	53	
IPI	2	10	29	29	41	.28
	3	19	54	25	36	.09
	>4	6	17	16	23	.67
**Bulky (≥7.5 sm)**	**Yes**	**21**	**60**	**24**	**34**	**.02**
	**No**	**14**	**40**	**46**	**68**	
Extranodal >1 site	Yes	27	77	50	71	.69
	No	8	23	20	29	
Bone marrow infiltration	Yes	7	20	17	24	.8
	No	28	80	53	76	
Lung involvement	Yes	7	20	15	21	.86
	No	28	80	55	79	
Gastric involvement	Yes	13	37	15	21	.13
	No	22	63	55	79	
Cell of origin	GCB	16	46	21	30	1.0
	Non‐GCB	19	54	27	39	
	No data	0	0	22	31	
DEL	Yes	13	37	21	30	.82
	No	22	63	31	44	
	No data	0	0	18	26	
**ICT**	**R‐CHOP**	**16**	**46**	**55**	**79**	**.004**
	**DA‐EPOCH‐R**	**19**	**54**	**15**	**21**	

Characteristic	Criteria	Upfront, *n* = 35	Control, *n* = 70	*p*‐value
Observation period (months)	Mean ± SDev	60.6 ± 22.4	62.5 ± 41.1	.78
	Median (range)	68 (19–82)	55 (6–143)	
Age (years)	Mean ± SDev	48.0 ± 11.7	48.9 ± 12.5	.53
	Median (range)	53 (18–63)	53 (22–64)	
LDH (U/l)	Mean ± SDev	385.3 ± 272.9	417.7 ± 240.6	.42
	Median (range)	295 (169–1450)	347 (145–1065)	
**Ki67 (%)**	**Mean ± SDev**	**81.7 ± 13.5**	**86.9 ± 14.1**	**.03**
	**Median (range)**	**82.5 (40–100)**	**90 (50–100)**	

*Note*: Bold indicates statistically significant value.

### 3.2 Clinical response

**FIGURE 2:**
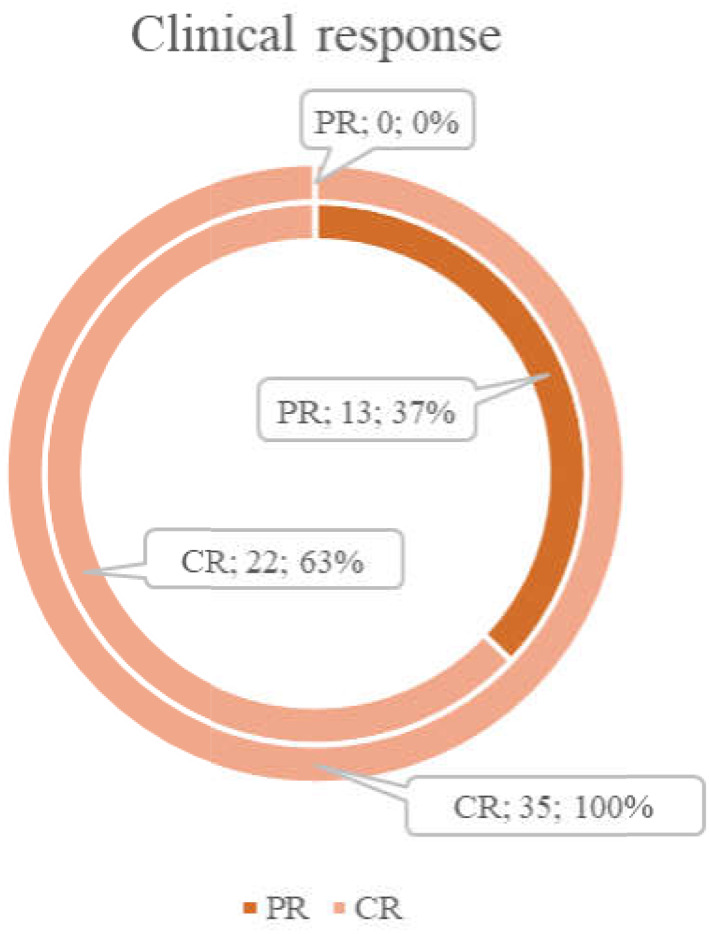
Clinical response in upfront group. Inner ring—response after ICT. Outer ring—response after upfront HDCT with auto‐HSCT.

There were 63% (22/35) CR in patients, who further enrolled in the upfront group and 100% (70/70) in the control group. After auto‐HSCT in the upfront group, the CR rate significantly increased (*p* = .0009) from 63% (22/35) to 100% (35/35) (Figure 2).

### 3.3 Relapse rate and cause of death

**FIGURE 3:**
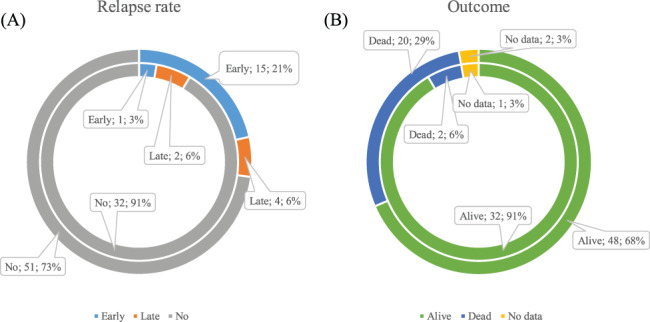
Between‐group comparisons. Inner ring–upfront group, *n* = 35. Outer ring—control group, *n* = 70. (A) Relapse rate; (B) Outcome.

A total of 3% (1/35) early (ER) and 6% (2/35) late (LR) relapses occurred in the upfront group (Figure 3A). All patients with ER and one patient with LR died from disease progression. One patient with LR underwent salvage ICT and survived (Figure 3B).

In the control group, 21% (15/70) ER and 6% LR (4/70) occurred (Figure 3A). In ER patients, the main cause of death was disease progression (*n* = 8). Two patients died from non‐DLBCL causes, and 3 patients from unknown reasons. One patient underwent salvage auto‐HSCT and is still alive, and the last one was lost in follow‐up. In LR patients, two died from disease progression, one from salvage HDCT complication (pneumonia), and one underwent auto‐HSCT in relapse and is still alive. In no relapse patients, two died from secondary malignancies, one from non‐oncological surgical intervention, and one from unknown reason (Figure 3B).

There was a significantly higher early relapse rate in the control group (1/35 vs. 15/70, *p* = .027) and a tendency in all relapse rate differences (3/35 vs. 19/70, *p* = .051). In the control group, early relapses occurred more frequently (15/19 vs. 4/19, *p* = .012). Early relapse rate depending on the response type did not demonstrate any significant differences (2/13 in PR and 1/22 in CR, *р* = .54 in the upfront group and 1/10 in PR vs. 18/60 in CR, *р* = .27 in the control group).

### 3.4 Survival analysis

#### 3.4.1 Progression‐free survival

**FIGURE 4:**
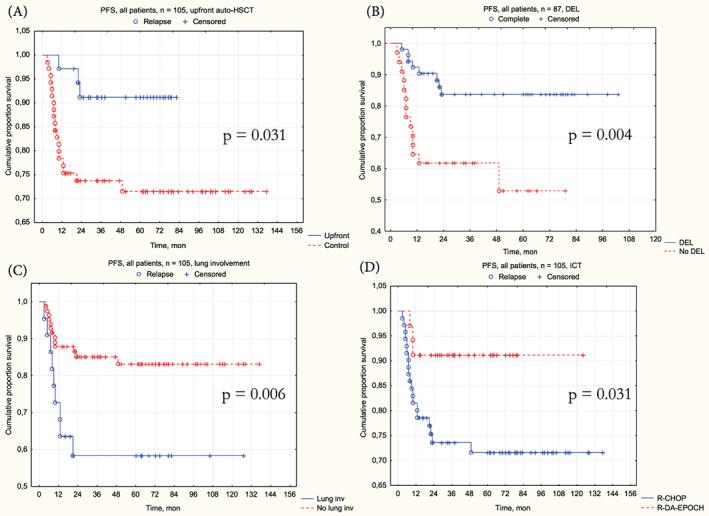
Progression‐free survival results. (A) PFS by treatment groups. (B) PFS by DEL. (C) PFS by lung involvement. (D) PFS by ICT.

The favor of upfront was demonstrated (Figure 4A) in 3‐year PFS–91.2% [95% CI 82.1%–100%] in upfront versus 73.6% [95% CI 63.8%–84.9%] in control, *p* = .018; HR = 0.26 [95% CI 0.08–0.89], *p* = .031.

Patients with double‐expressor lymphomas (DEL) demonstrated (Figure 4B) worse 3‐year PFS–61.6% [95% CI 47.2%–80.4%] with DEL versus 83.7% [95% CI 74.0%–94.8%] without DEL, *p* = .004; HR = 3.42 [95% CI 1.43–8.18], *p* = .006. Lung involvement was another significant factor (Figure 4C), worsening the 3‐year PFS–58% [95% CI 40.4%–83.4%] in patients with extranodal lung lesions versus 85.1% [95% CI 77.7%–93.3%] without it, *p* = .004; HR = 3.42 [95% CI 1.43–8.18], *p* = .006.

Patients, treated by DA‐EPOCH‐R demonstrated (Figure 4D) a trend of superior 3‐year PFS–91.2% [95% CI 82.1%–100%] in upfront versus 73.6% [95% CI 63.8%–84.9%] in control, *p* = .031, but HR was borderline—0.3 [95% CI 0.09–1.00], *p* = .05.

Multivariate PFS analysis demonstrated the benefit in 3‐year PFS (Table 2, Data S5) by upfront auto‐HSCT (HR = 0.26, 95% CI [0.07–0.9], *p* = .033) and DA‐EPOCH‐R (HR = 0.26, 95% CI [0.07–0.94], *p* = .041). Gastric lesions also demonstrated a positive impact on PFS (HR = 0.19, 95% CI [0.05–1.87], *p* = .033). The presence of DEL was the only factor, that demonstrated a significant negative impact (HR = 4.22, 95% CI [1.72–10.37], *p* = .001).

**TABLE 2:** Multivariate PFS analysis in all patients, *n* = 87.

Factor	Univariate		Multivariate	
	HR (95% CI)	*p*	HR (95% CI)	*p*
**Study group**	**0.19 (0.05–0.63)**	**.007**	**0.26 (0.07–0.9)**	**.033**
**Control (*n* = 52)**				
**Upfront (*n* = 35)**				
**DEL**	**3.41 (1.43–8.16)**	**.006**	**4.22 (1.72–10.37)**	**.001**
**No (*n* = 53)**				
**Yes (*n* = 34)**				
**Lung lesions**	**2.6 (1.11–6.1)**	**.028**	1.36 (0.54–3.43)	.521
**No (*n* = 67)**				
**Yes (*n* = 20)**				
**Gastric lesions**	**0.2 (0.05–0.84)**	**.024**	**0.19 (0.05–1.87)**	**.033**
**No (*n* = 61)**				
**Yes (*n* = 26)**				
**ICT**	**0.23 (0.07–0.79)**	**.019**	**0.26 (0.07–0.94)**	**.041**
**R‐CHOP (*n* = 55)**				
**DA‐EPOCH‐R (*n* = 32)**				

There were too few relapses in the upfront group (*n* = 3) to determine any significant factors in PFS. The only factor, that demonstrated negative PFS significance in the control group (Table 3, Data S6) was DEL (HR = 5.62, 95% CI [1.97–16.01], *p* = .001).

**TABLE 3:** Multivariate PFS analysis in the control group, *n* = 52.

Factor	Univariate		Multivariate	
	HR (95% CI)	*p*	HR (95% CI)	*p*
**DEL**	**6.18 (2.2–17.32)**	**.001**	**5.62 (1.97–16.01)**	**.001**
**No (*n* = 31)**				
**Yes (*n* = 21)**				
**Lung lesions**	**3.26 (1.32–8.07)**	**.011**	2.13 (0.81–5.59)	.123
**No (*n* = 39)**				
**Yes (*n* = 13)**				
Gastric lesions	0.28 (0.06–1.2)	.087	0.36 (0.08–1.66)	.189
No (*n* = 39)				
Yes (*n* = 13)				

#### 3.4.2 Overall survival

**FIGURE 5:**
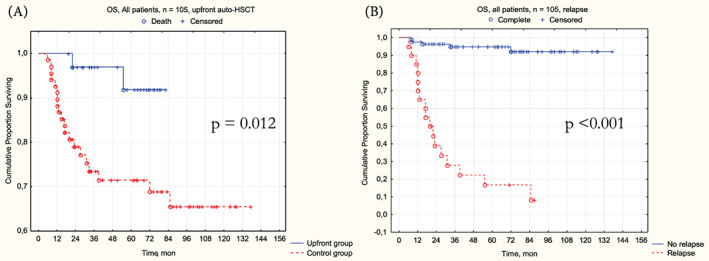
Overall survival results. (A) OS by treatment groups; (B) OS by relapse occurrence.

Patients in the upfront group demonstrated significantly higher (Figure 5A) 3‐year OS–97.1% [95% CI 91.8%–100%] in upfront versus 75% [95% CI 65.4%–86.3%] in control, *p* = .012; HR = 0.18 [95% CI 0.04–0.78, *p* = .022]. Another significant factor for 3‐year OS was the relapse occurrence (Figure 5B)–97.6% [95% CI 94.3%–100%] without relapse versus 30.7% [95% CI 16.1%–58.3%] with relapse, *p* < .001; HR = 30.4 [95% CI 10.1–91.5], *p* < .001.

Multivariate OS analysis confirmed the significant characteristics–upfront auto‐HSCT was associated with better 3‐year OS (HR = 0.11, 95% CI [0.02–0.63], *p* = .013) and relapse with worse 3‐year OS (HR = 39.38, 95% CI [7.93–195.4], *p* < .001). Interestingly, the weight loss, as a component of B‐symptoms, showed its worsening prognostic significance (HR = 3.11, 95% CI [1.05–9.19], *p* = .04) in the multivariate setting (Table 4, Data S7).

**TABLE 4:** Multivariate OS analysis in all patients, *n* = 80.

Factor	Univariate		Multivariate	
	HR (95% CI)	*p*	HR (95% CI)	*p*
**Study group**	**0.14 (0.03–0.63)**	**.01**	**0.11 (0.02–0.63)**	**.013**
**Control (*n* = 47)**				
**Upfront (*n* = 33)**				
DEL	2.5 (0.97–6.47)	.074	0.36 (0.11–1.21)	.098
No (*n* = 48)				
Yes (*n* = 32)				
Gastric lesions	0.25 (0.06–1.07)	.062	0.56 (0.12–2.69)	.453
No (*n* = 56)				
Yes (*n* = 24)				
ICT	0.31 (0.09–1.06)	.062	1.37 (0.34–5.42)	.658
CHOP‐R (*n* = 49)				
DA‐EPOCH‐R (*n* = 31)				
**Weight loss**	2.11 (0.79–5.65)	.137	**3.11 (1.05–9.19)**	**.04**
**No (*n* = 62)**				
**Yes (*n* = 18)**				
**Relapse**	**25.3 (7.27–88.2)**	**<.001**	**39.38 (7.93–195.4)**	**<.001**
**No (*n* = 61)**				
**Yes (*n* = 19)**				

There were too few deaths (*n* = 2) in the upfront group to determine any significant factors in 3‐year OS. In the control group, negative 3‐year OS impact was demonstrated by relapse occurrence (HR = 24.06, 95% CI [5.73–101], *p* < .001) only (Table 5, Data S8).

**TABLE 5:** Multivariate OS analysis in the control group, *n* = 51.

Factor	Univariate		Multivariate	
	HR (95% CI)	*p*	HR (95% CI)	*p*
**DEL**	**2.6 (1.03–6.54)**	**.043**	0.48 (0.17–1.35)	.162
**No (*n* = 30)**				
**Yes (*n* = 21)**				
**Adrenal lesions**	**4.85 (1.34–17.59)**	**.016**	2.97 (0.78–11.25)	.109
**No (*n* = 47)**				
**Yes (*n* = 4)**				
**Relapse**	**15.9 (4.56–55.4)**	**<.001**	**24.06 (5.73–101)**	**<.001**
**No (*n* = 32)**				
**Yes (*n* = 19)**				

## 4 DISCUSSION

The role of upfront auto‐HSCT in DLBCL remains controversial. EBMT (level of evidence 1)^28^ and NCCN (level of evidence 2B) guidelines^29^ suggest it for young high‐risk patients or as a clinical trial option. ASTCT recommends upfront auto‐HSCT only with *c‐myc* and *bcl‐2* and/or *bcl‐6* rearrangements (high‐grade lymphomas).^30^

These statements are based on the results of RCT. The improvement of 2‐year FFS (*p* = .038), achieved in randomized, phase 3, DLCL04 trial,^12^ did not translate into OS benefit (*p* = .9). That can be associated with the effect of second‐line HDCT with auto‐HSCT,^31^ which was performed in more than 50% of relapsed patients in non‐upfront groups or with a low inclusion rate (26%) of high‐risk (aaIPI = 3) patients. High‐risk patients also failed to demonstrate 2‐year FFS improvement (*p* = .16) No difference in efficacy of intensified induction ICT wasn't shown either.

In NCT00355199,^13^ the improvement of 3‐year DFS (*p* = .03) was temporal and vanished in 5 years (*p* = .14). Also, there was no benefit in OS (*p* = .64). The primary objective of EFS improvement was not achieved, however, only 71% of patients in the R‐HDS group underwent the ASCT because of a higher rate of adverse events.

SWOG‐S7904 trial^19^ demonstrated a significant improvement of 2‐year PFS (*p* = .001) and 2‐year OS (*p* = .001) in high‐intermediate/high‐risk and DEL patients by the separate subset analysis.^32^ Also, 2‐year PFS was significantly higher (*p* = .005) even without risk stratification. But, 29.6% (75/253) randomized patients were treated by CHOP and not R‐CHOP and this compromises the overall result interpretation.

On the other hand, DSHNHL 2002‐1 trial demonstrated only increased toxicity in the HDCT group without survival benefit.^20^ Moreover, the R‐CHOEP‐14 group demonstrated better survival than R‐MegaCHOEP patients with repetitive auto‐HSCT.

Meta‐analyses^17^, ^18^ did not prove the OS benefit from upfront auto‐HSCT. The first one, by Epperla et al.,^17^ demonstrated only increased toxicity (*р* < .001) in HDCT patients without any PFS (*р* = .09) or OS (*р* = .22) difference between groups, even with high aaIPI limitation.

PFS benefit in high‐intermediate and high‐risk DLBCL patients (*р* = .0002) and a trend to OS (*р* = .06) improvement were found only in analysis by Ma et al.,^18^ which included retrospective trials. Also, this meta‐analysis showed that CR patients after upfront auto‐ASCT have a better OS (*р* = .008), but not high‐risk patients (*р* = .2). This statement was approved by retrospective analysis.

Our study demonstrated the positive role of upfront auto‐HSCT in the survival of stage IV DLBCL NOS high/high‐intermediate IPI risk patients.

The key point of this study was patient selection. Most previous trials included all types of B‐cell and even T‐cell lymphomas.^19^ Two randomized trials with positive results of upfront auto‐HSCT included more than 90% of DLBCL in each group.^12^, ^13^ We included only NOS subtype, the most common variant of DLBCL,^5^ to decrease the selection bias.

The decision to perform upfront auto‐HSCT in the described group (*n* = 105) was made by MDT case by case and depended mostly on the initial state, patient preference, and current load of the HSCT unit. Stage IV was the negative prognostic factor^15^, ^16^ in previous trials, and high/high‐intermediate patients had the highest relapse rate and the worst survival.^5^

Trial arms were completely balanced in all characteristics. A higher proportion of bulky disease in the upfront group is not clinically significant because it does not always correspond with high metabolic tumor volume, calculated by PET‐CT.^29^, ^33^

In this trial, PET‐CT was done once in 71% (25/35) upfront patients and 41% (29/70) of control patients. PET scans before and after the treatment had only 23% (8/35) upfront patients and 17% (12/70) control patients. The number of remissions, evaluated only by CT and biopsy was 40% (14/35) in the upfront and 59% (41/70) in the control group. Low incidence of PET resulted from the scarce of original PET scans and the lack of PET in Russia in the early 2010's.

The achievement of CR is the main goal of treatment. Partial responses are considered treatment failure and recommended to manage as a refractory disease.^34^ The control group was combined from CR to compare upfront HDCT with auto‐HSCT only with completely treated patients.

The association of DEL and inferior survival (*p* = .001) was the anticipated result because many trials have shown it before.^35^, ^36^, ^37^ The need and the benefit of treatment intensification in DEL/DHL patients was demonstrated in retrospective trials. In trial by Lansburg et al.,^38^ patients with intensive (DA‐EPOCH‐R, R‐HyperCVAD, R‐CODOX‐M/IVAC) ICT experienced superior rates in 3‐year RFS, compared with R‐CHOP (75% vs. 51% respectively, *p* = .003). Upfront HDCT with auto‐HSCT after R‐CHOP demonstrated the same benefit above R‐CHOP only (3‐year RFS 75% vs. 51% respectively, *p* = .001).

No difference was estimated between R‐CHOP/upfront and intensive ICT (3‐year RFS 75% vs. 86% respectively, *p* = .51) or in R‐CHOP/upfront versus intensive/upfront (3‐year RFS 75% vs. 91% respectively, *p* = .47). There were no differences in OS either. Authors suggested, that proceeding with consolidative autoSCT in DHL patients with CR may be justifiable as the result of the relatively poor survival outcomes for R‐CHOP/non‐autoSCT patients demonstrated in this series.

Another trial targeted DEL DLBCL patients with elevated LDH treated by upfront HDCT.^39^ The 5‐year OS and PFS for DEL patients were 76.1% and 77.8%, respectively (*p* = .978), and 81.8% and 80.3%, respectively (*p* = .841) for non‐DEL patients. Authors proposed, that upfront HDCT with auto‐HSCT overcomes the poor prognostic effect of DEL on the non‐GCB DLBCL.

In our trial, DEL significantly decreased 3‐year PFS (*p* = .001). Because of high cost, FISH was performed partially and we could not exclude double‐ or triple‐hit lymphomas. However, the incidence of them is about 10% in all DLBCL cases,^40^ and our DEL amount was relatively small (*n* = 34). C‐MYC (*p* = .64), BCL2 (*p* = .67), and BCL6 (*p* = .75) mono expressions without DEL did not affect survival.

The non‐GCB COO subtype is usually associated with poor prognosis in DLBCL.^41^, ^42^ The different roles of molecular classification, according to upfront HDCT in DLBCL with elevated LDH were reported in retrospective trials.^14^, ^22^ In our trial, non‐GCB subtype showed only tendency to increase the relapse rate (*p* = .11) and decrease PFS (*p* = .102) with no impact on OS (*p* = .48). This result is questionable and should be investigated further.

A high proliferation index (Ki67) is generally considered a negative prognostic marker in B‐cell lymphomas. A meta‐analysis,^43^ RCT (Ki67 threshold 75% in subgroup with IPI 2–3)^44^ and a couple of retrospective trials (different thresholds up to 85%)^45^, ^46^ demonstrated the significance of high Ki67 as a prognostic marker in rituximab treated DLBCL. We tried to use various thresholds and a continuous variable (*p* = .5) to determine the possible prognostic significance but no correlation with survival characteristics was found in all comparisons.

The high amount of CD3(+) tumor‐infiltrating lymphocytes (TILs), assessed by immunohistochemistry (IHC), is associated with favorable outcomes in advanced stage DLBCL,^47^ especially among aggressive subtypes of DLBCL, like testicular^48^ and skin^49^ in retrospective trials. In our group, TILs were measured only in 33% (35/105) patients and we did not find any significant correlations between CD3(+) TILs and OS (*p* = .22) or PFS (*p* = .32).

It is an interesting result about the superiority of DA‐EPOCH‐R over R‐CHOP in our trial because randomized trials did not find any survival benefit in advanced‐stage DLBCL, treated by DA‐EPOCH‐R.^50^ However, there are retrospective trials on DEL DLBCL young patient populations, where DA‐EPOCH‐R demonstrated better results.^51^ In our trial, treatment groups were statistically different in DA‐EPOCH‐R percentage (*p* = .004). The univariate PFS analysis did not show DA‐EPOCH‐R benefit (*p* = .46) in the control group. The event incidence in the upfront group was too low to verify any significant characteristic. To determine the role of DA‐EPOCH‐R in this subgroup a larger trial with a different design should be done.

Another controversial result was the influence of unexplained loss of >10% of body weight (*n* = 17) over the past 6 months (B‐symptom) on OS. B‐symptoms aren't a standard pretreatment prognostic factor in DLBCL. There are only retrospective trials, approving that patients with B‐symptoms are more likely to have shorter event‐specific survival^52^ or demonstrate survival impact in selected subgroups.^53^ None of this kind of trials targeted B‐symptom‐associated weight loss as an independent risk factor. By now, this result should be considered a calculation artifact.

High level of LDH associated with poor prognosis and predicts survival in DLBCL patients.^54^ The IPI modifications, like aaIPI^55^ or CNS‐IPI^56^ better distinguish risk groups and are used in special conditions. According to the NCCN‐IPI RCT,^57^ the LDH ratio stratification can play a prognostic role in DLBCL. We used our LDH means (*p* = .52) and medians (*p* = .43) as thresholds but none of them were significantly affected PFS. This result may be associated with relatively small group (*n* = 105). For example, NCCN‐IPI RCT^57^ included 2788 patients.

This study had several limitations. Firstly, it was retrospective, and the patient selection wasn't randomized. However, strict selection criteria were applied, therefore trial arms were completely balanced. Secondly, we did not use the gene expression profile (GEP) based molecular classification because of the absence of GEP in our institution, and were limited in FISH for financial reasons. Also, there were several missing IHC data, that limited the inclusion of IHC parameters in calculations. And thirdly, because of limited PET data, modern PET‐based predictors like metabolic tumor volume or tumor lesion glycolysis were not analyzed.

In conclusion, the upfront auto‐HSCT is still under investigation. Further trials are needed to better select the target group of this treatment option.

## 5 CONCLUSION

In this trial, upfront patients demonstrated significantly better 3‐year OS and PFS. It is a relatively small period, and further follow‐up is needed to confirm the sustainability of our result. The significant difference in early relapse rate and the same tendency in all relapse rates in favor of upfront auto‐HSCT is promising.

We suggest, that upfront auto‐HSCT can serve as a familiar and adequate option to significantly improve a prognosis in young high‐risk patients with DEL DLBCL. This clinical implication does not require special additions and can be used in any HSCT unit. However, prospective randomized trials are needed to confirm the significance of DEL in this cohort.

In summary, upfront HDCT with auto‐HSCT can be a possible treatment option in DLBCL NOS, age 18–65, stage IV, IPI ≥2 patients.

## AUTHOR CONTRIBUTIONS

**Aleksei K. Koviazin:** Conceptualization (lead); data curation (lead); formal analysis (lead); investigation (lead); methodology (lead); project administration (lead); resources (lead); software (lead); supervision (lead); validation (lead); visualization (lead); writing – original draft (lead); writing – review and editing (lead). **Larisa V. Filatova:** Conceptualization (equal); data curation (supporting); formal analysis (supporting); investigation (equal); methodology (equal); project administration (supporting); resources (supporting); supervision (equal); validation (supporting). **Ilia S. Zyuzgin:** Data curation (supporting); project administration (supporting); resources (supporting); software (supporting). **Anna S. Artemyeva:** Data curation (equal); investigation (equal); resources (supporting). **Ilia L. Poliatskin:** Data curation (equal); investigation (equal); resources (supporting). **Darya S. Burda:** Data curation (equal); investigation (equal); resources (supporting). **Stanislav A. Volchenkov:** Resources (supporting). **Svetlana S. Elkhova:** Resources (supporting). **Tatiana Yu. Semiglazova:** Data curation (supporting); methodology (supporting); resources (supporting).

## CONFLICT OF INTEREST STATEMENT

The authors have stated explicitly that there are no conflicts of interest in connection with this article.

## ETHICS STATEMENT

All procedures performed in studies involving human participants were by the ethical standards of the institutional and/or national research committee and with the 1964 Helsinki declaration and its later amendments or comparable ethical standards.

## Supporting information


**Data S1**. Supporting Information.

**Data S2**. Supporting Information.

**Data S3**. Supporting Information.

**Data S4**. Supporting Information.

**Data S5**. Supporting Information.

**Data S6**. Supporting Information.

**Data S7**. Supporting Information.

**Data S8**. Supporting Information.

## Data Availability

Data sharing is not applicable to this article as no new data were created or analyzed in this study.
