# Supplementary material for: The significance of upfront autologous stem cell transplantation for high‐intermediate/high‐risk stage IV diffuse large B‐cell lymphoma
Source: Cancer Rep (Hoboken). 2023 Feb 28;6(4):e1786. doi: 10.1002/cnr2.1786 (PMC10075296; doi:10.1002/cnr2.1786)
Supplement: Supplementary file 1 — Data S1. Supporting Information. [file CNR2-6-e1786-s004.pdf]

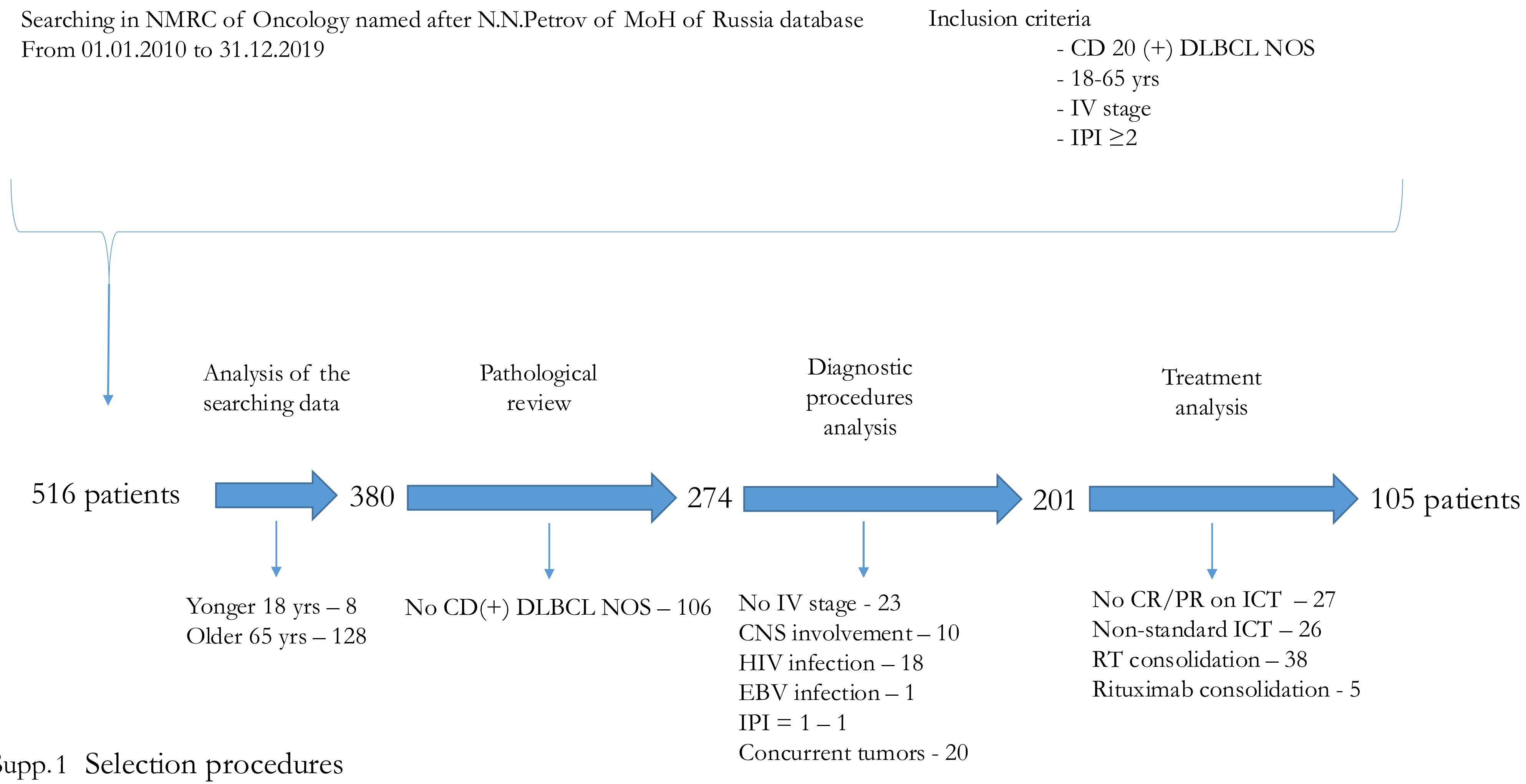

Supp.1 Selection procedures
