# Supplementary material for: The significance of upfront autologous stem cell transplantation for high‐intermediate/high‐risk stage IV diffuse large B‐cell lymphoma
Source: Cancer Rep (Hoboken). 2023 Feb 28;6(4):e1786. doi: 10.1002/cnr2.1786 (PMC10075296; doi:10.1002/cnr2.1786)
Supplement: Supplementary file 2 — Data S2. Supporting Information. [file CNR2-6-e1786-s001.pdf]

Mann-Whitney and normality tests

Independent Samples T-Test

		Statistic	p	Mean difference	SE difference	95% Confidence Interval	
						Lower	Upper
Ki67	Mann-Whitney U	802	0.031	-5.00		-10.00	-4.66e−6
LDH	Mann-Whitney U	711	0.416	31.00		-40.00	115.00
Age	Mann-Whitney U	1133	0.531	1.00		-3.00	5.00
OS	Mann-Whitney U	1184	0.780	-2.00		-16.00	14.00
PFS	Mann-Whitney U	1148	0.603	-4.00		-20.00	14.00

Assumptions

Tests of Normality

		statistic	p
Ki67	Shapiro-Wilk	0.911	< .001
	Kolmogorov-Smirnov	0.1944	0.001
	Anderson-Darling	3.01	< .001
LDH	Shapiro-Wilk	0.825	< .001
	Kolmogorov-Smirnov	0.1751	0.012
	Anderson-Darling	4.32	< .001
Age	Shapiro-Wilk	0.899	< .001
	Kolmogorov-Smirnov	0.1638	0.007
	Anderson-Darling	3.70	< .001
OS	Shapiro-Wilk	0.957	0.002
	Kolmogorov-Smirnov	0.1074	0.177
	Anderson-Darling	1.30	0.002
PFS	Shapiro-Wilk	0.948	< .001
	Kolmogorov-Smirnov	0.0965	0.283
	Anderson-Darling	1.41	0.001

Note. Additional results provided by *moretests*

Group Descriptives

	Group	N	Mean	Median	SD	SE
Ki67	Control	64	81.7	82.5	13.5	1.68
	Upfront	34	86.9	90.0	14.1	2.43
LDH	Control	55	417.8	347.0	240.7	32.46
	Upfront	29	385.3	295.0	272.9	50.68
Age	Control	70	48.9	53.0	12.6	1.50
	Upfront	35	48.0	53.0	11.8	1.99
OS	Control	70	62.5	55.0	41.2	4.92
	Upfront	35	60.6	68.0	22.5	3.80
PFS	Control	70	58.1	48.0	44.1	5.27
	Upfront	35	57.8	60.0	24.6	4.16
