# Supplementary material for: The significance of upfront autologous stem cell transplantation for high‐intermediate/high‐risk stage IV diffuse large B‐cell lymphoma
Source: Cancer Rep (Hoboken). 2023 Feb 28;6(4):e1786. doi: 10.1002/cnr2.1786 (PMC10075296; doi:10.1002/cnr2.1786)
Supplement: Supplementary file 3 — Data S3. Supporting Information. [file CNR2-6-e1786-s003.docx]

| Explanatory | Levels | All | HR (Univariable) | p-value | Explanatory | Levels | All | HR (Univariable) | p-value |
| --- | --- | --- | --- | --- | --- | --- | --- | --- | --- |
| **Study group** | **Control**  **Upfront** | **70**  **35** | **-**  **0.27 (0.08 – 0.91)** | **0.034** | Extranodal  lesion | 1  ≥ 2 | 28  77 | -  1.31 (0.48 – 3.55) | 0.595 |
| Cell of origin | GCB  Non-GCB | 33  50 | -  2.94 (0.83 – 10.4) | 0.096 | Bone marrow  lesion | No  Yes | 81  24 | -  0.95 (0.35 – 2.58) | 0.925 |
| **DEL** | **No**  **Yes** | **52**  **35** | **-**  **3.41 (1.43 – 8.16)** | **0.006** | **Lung**  **lesion** | **No**  **Yes** | **83**  **22** | **-**  **2.91 (1.24 – 6.82)** | **0.014** |
| Sex | Female  Male | 55  50 | -  1.13 (0.49 – 2.60) | 0.776 | Adrenal  lesion | No  Yes | 93  11 | -  1.52 (0.45 – 5.14) | 0.501 |
| IPI | < 3  ≥ 3 | 39  66 | -  1.65 (0.64 – 4.21) | 0.297 | B-symptoms | No  Yes | 55  50 | -  0.64 (0.27 – 1.52) | 0.306 |
| ECOG | < 2  ≥ 2 | 88  17 | -  1.17 (0.39 – 3.44) | 0.782 | B-symptoms  Temperature | No  Yes | 74  23 | -  0.58 (0.17 – 1.99) | 0.387 |
| Bulky | No  Yes | 60  45 | -  0.6 (0.24 – 1.46) | 0.259 | B-symptoms  Night swear | No  Yes | 76  21 | -  1 (0.33 – 3.02) | 0.998 |
| ICT | R-CHOP  R-DA-EPOCH | 71  34 | -  0.3 (0.09 – 1.01) | 0.051 | B-symptoms  Weight loss | No  Yes | 78  19 | -  1.19 (0.88 – 6.13) | 0.761 |
| Response | PR  CR | 13  92 | -  1.56 (0.36 – 6.68) | 0.548 | Gastric  lesion | No  Yes | 77  28 | -  0.24 (0.06 – 1.03) | 0.055 |

Supplement 3: Univariable analysis of all categorical characteristics in PFS

Statistically significant results are in bold.
