# Supplementary material for: The significance of upfront autologous stem cell transplantation for high‐intermediate/high‐risk stage IV diffuse large B‐cell lymphoma
Source: Cancer Rep (Hoboken). 2023 Feb 28;6(4):e1786. doi: 10.1002/cnr2.1786 (PMC10075296; doi:10.1002/cnr2.1786)
Supplement: Supplementary file 4 — Data S4. Supporting Information. [file CNR2-6-e1786-s005.docx]

| Explanatory | Levels | All | HR (Univariable) | p-value | Explanatory | Levels | All | HR (Univariable) | p-value |
| --- | --- | --- | --- | --- | --- | --- | --- | --- | --- |
| **Study group** | **Control**  **Upfront** | **70**  **35** | **-**  **0.18 (0.04 – 0.78)** | **0.022** | Extranodal  lesion | 1  ≥ 2 | 28  77 | -  1.08 (0.42 – 2.76) | 0.877 |
| Cell of origin | GCB  Non-GCB | 33  50 | -  1.52 (0.52 – 4.48) | 0.448 | Bone marrow  lesion | No  Yes | 81  24 | -  0.52 (0.16 – 1.77) | 0.3 |
| DEL | No  Yes | 52  35 | -  2.17 (0.91 – 5.17) | 0.081 | Lung  lesion | No  Yes | 83  22 | -  1.89 (0.77 – 4.64) | 0.164 |
| Sex | Female  Male | 55  50 | -  1.35 (0.58 – 3.12) | 0.484 | Adrenal  lesion | No  Yes | 93  11 | -  1.56 (0.46 – 5.28) | 0.476 |
| IPI | < 3  ≥ 3 | 39  66 | -  1.4 (0.57 – 3.44) | 0.463 | B-symptoms | No  Yes | 55  50 | -  1.04 (0.45 – 2.42) | 0.925 |
| ECOG | < 2  ≥ 2 | 88  17 | -  1.26 (0.43 – 3.73) | 0.677 | B-symptoms  Temperature | No  Yes | 74  23 | -  0.59 (0.17 – 2.03) | 0.403 |
| Bulky | No  Yes | 60  45 | -  0.64 (0.26 – 1.57) | 0.326 | B-symptoms  Night swear | No  Yes | 76  21 | -  1.13 (0.37 – 3.44) | 0.824 |
| ICT | R-CHOP  R-DA-EPOCH | 71  34 | -  0.33 (0.1 – 1.12) | 0.076 | B-symptoms  Weight loss | No  Yes | 78  19 | -  2.32 (0.88 – 6.13) | 0.09 |
| Response | PR  CR | 13  92 | -  1.48 (0.35 – 6.34) | 0.597 | **Relapse** | **No**  **Yes** | **83**  **22** | **-**  **30.4 (10.1 – 91.5)** | **<0.001** |

Supplement 4: Univariable analysis of all categorical characteristics in OS

Statistically significant results are in bold.
