# Supplementary figures and images for: The significance of upfront autologous stem cell transplantation for high‐intermediate/high‐risk stage IV diffuse large B‐cell lymphoma
Source: Cancer Rep (Hoboken). 2023 Feb 28;6(4):e1786. doi: 10.1002/cnr2.1786 (PMC10075296; doi:10.1002/cnr2.1786)

# Hazard ratio

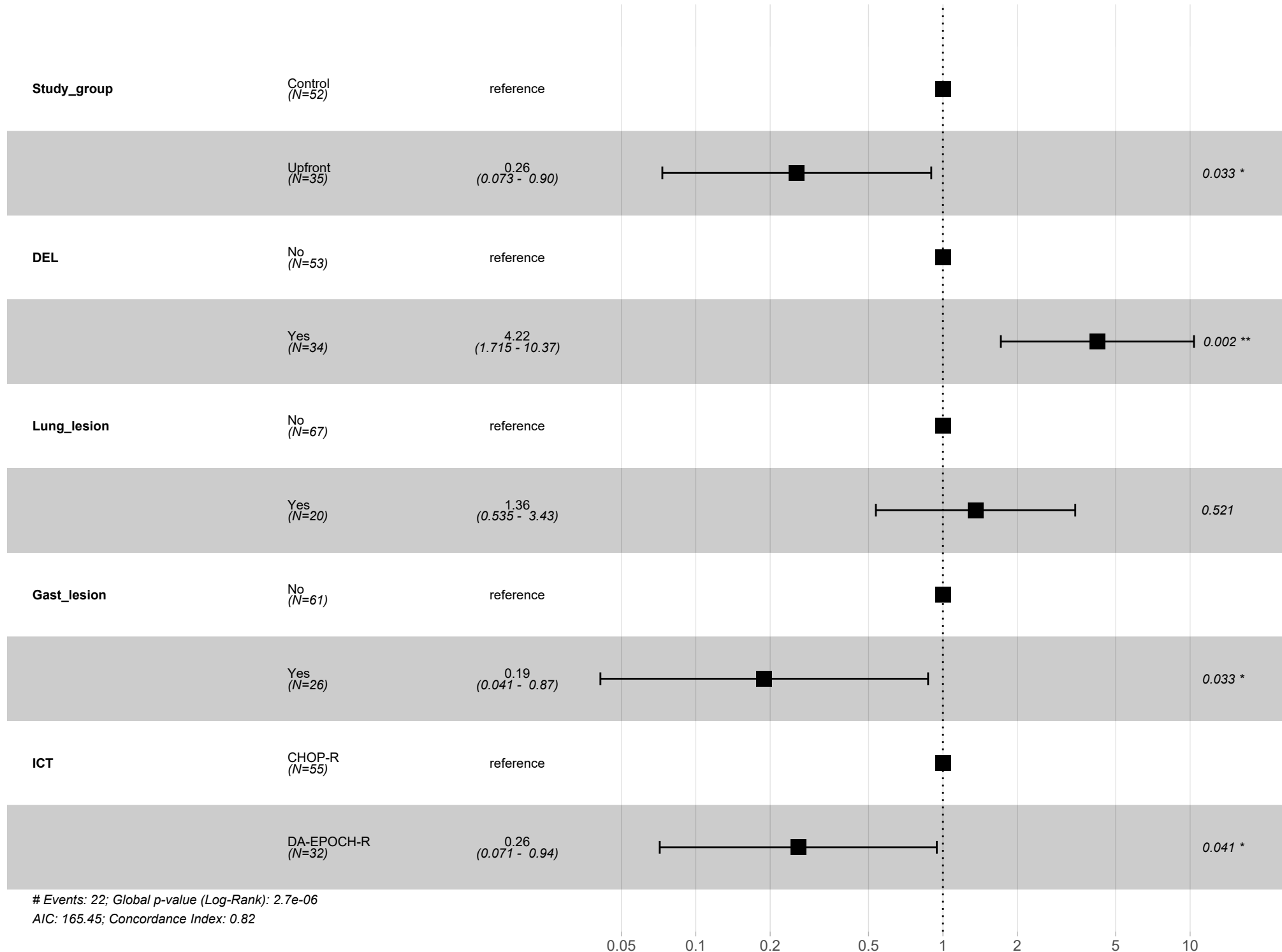

Supplement: Supplementary file 5 — Data S5. Supporting Information. [file CNR2-6-e1786-s006.pdf]

# Hazard ratio

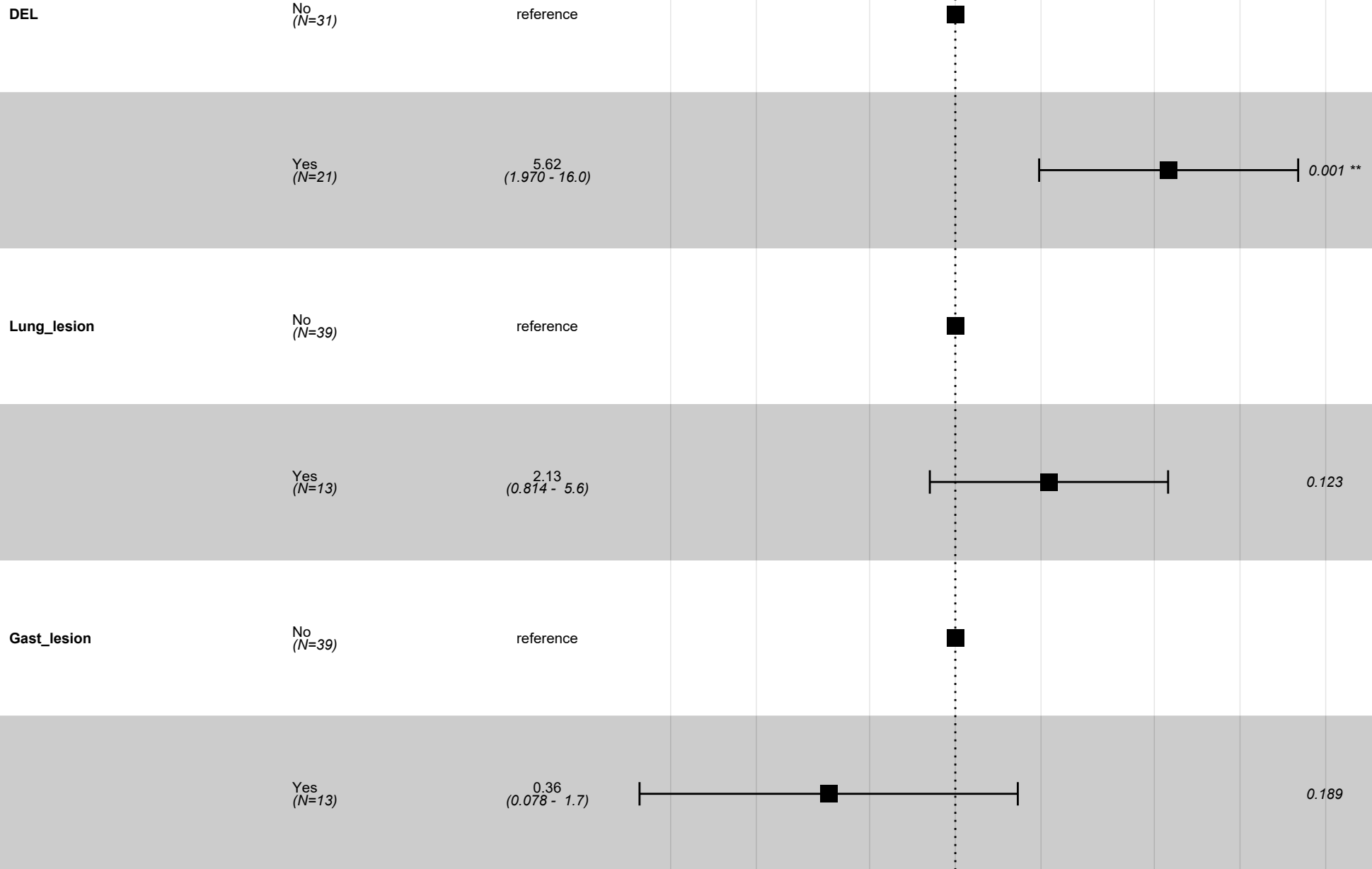

# Events: 19; Global p-value (Log-Rank): 0.00012  
 AIC: 126.25; Concordance Index: 0.8

0.1 0.2 0.5 1 2 5 10 20

Supplement: Supplementary file 6 — Data S6. Supporting Information. [file CNR2-6-e1786-s002.pdf]

# Hazard ratio

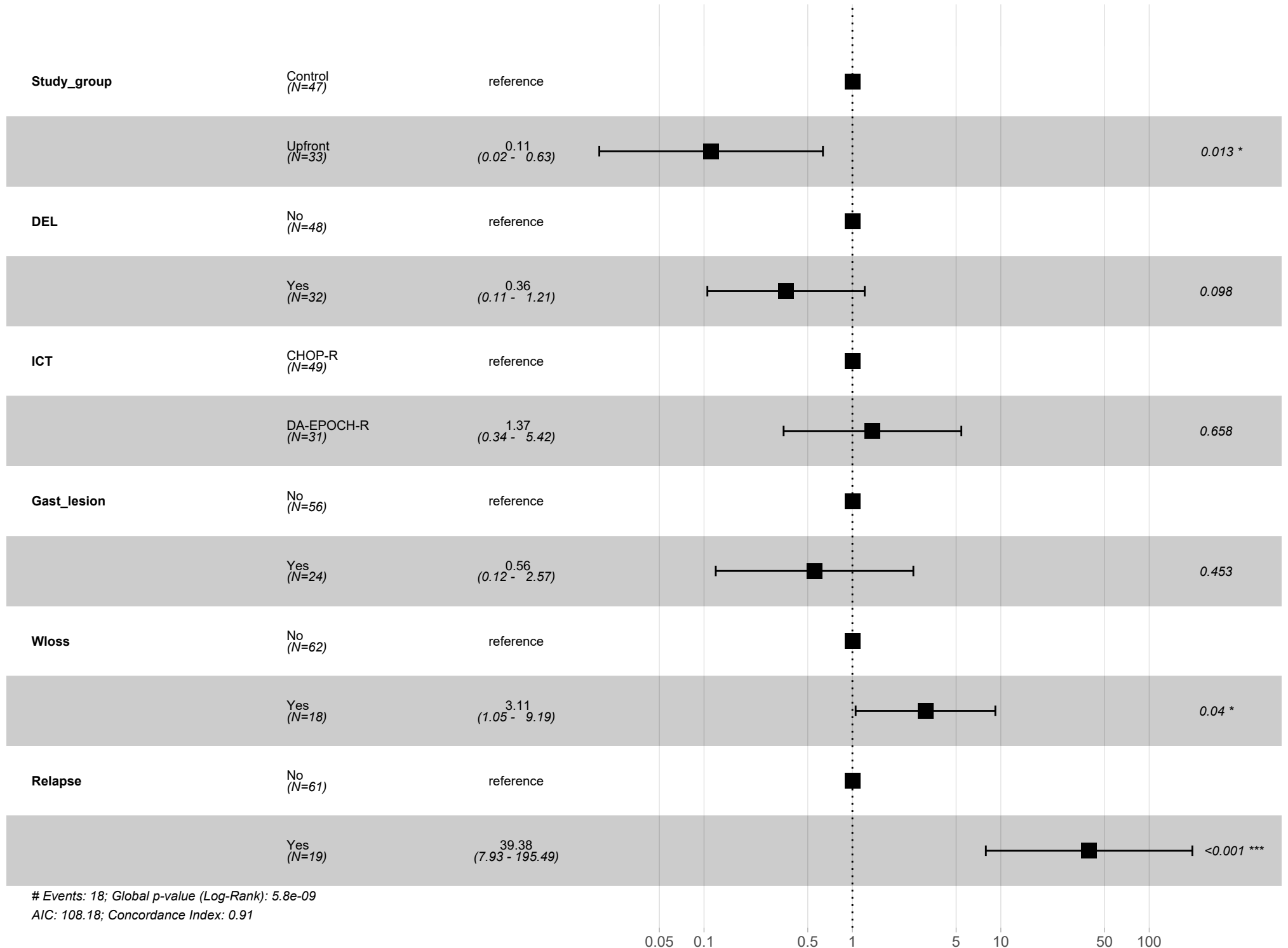

Supplement: Supplementary file 7 — Data S7. Supporting Information. [file CNR2-6-e1786-s008.pdf]

# Hazard ratio

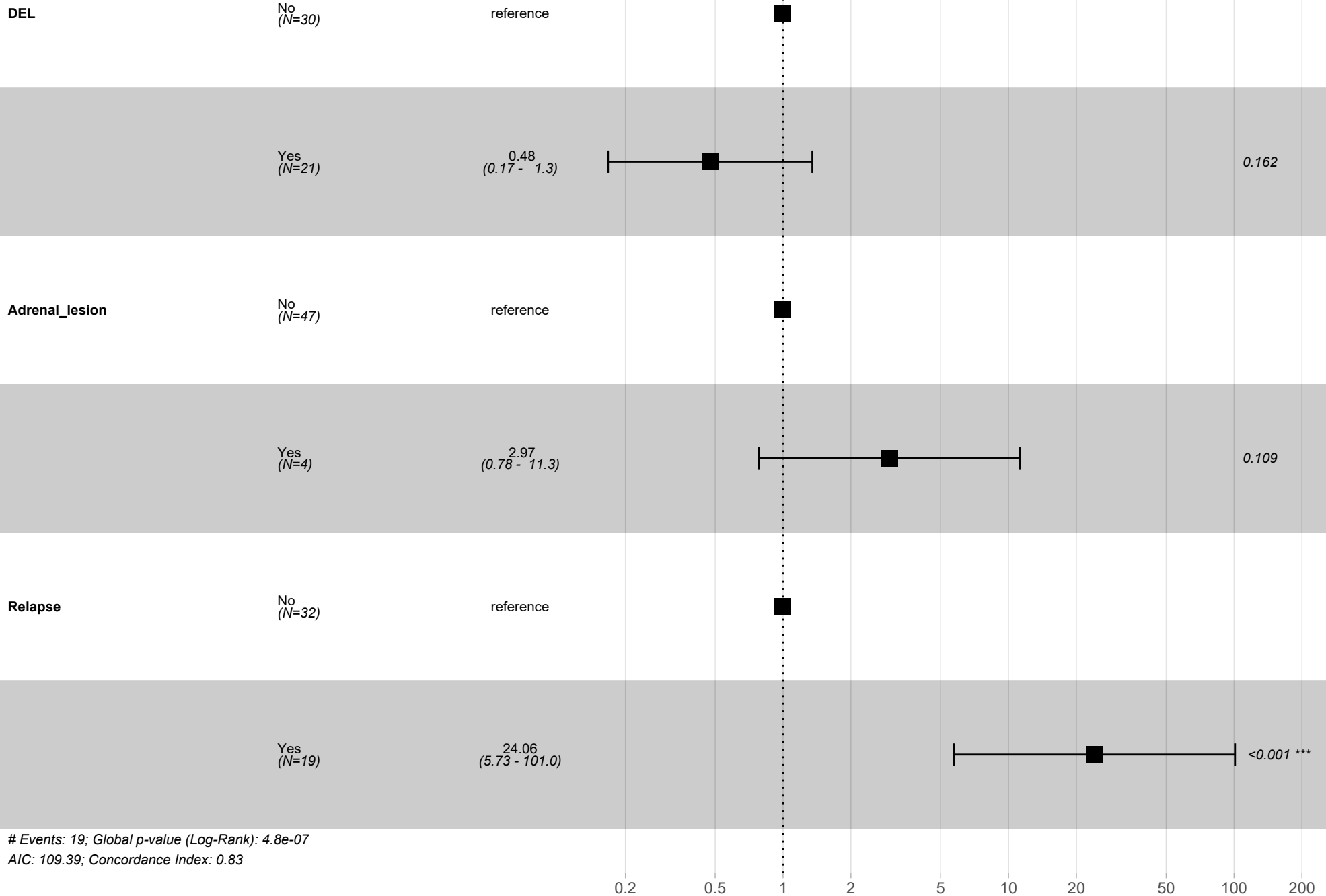

Supplement: Supplementary file 8 — Data S8. Supporting Information. [file CNR2-6-e1786-s007.pdf]
